# CT characterization of idiopathic inflammatory myopathy-associated interstitial lung disease: frontiers to strengthen diagnostic accuracy

**DOI:** 10.36416/1806-3756/e20250332

**Published:** 2025-11-19

**Authors:** Kadir Burak Akgün, Antonio M Esquinas

**Affiliations:** 1. Chest Diseases Department, Hatay Mustafa Kemal University, Hatay, Turkey.; 2. Intensive Care Unit Hospital Meseguer. NIV in ICU Group. Biomedical Research Institute Pascual Parrilla-IMIB-Murcia, Spain.

We read with great interest the article by Oliveira Filho et al. entitled “Clinical, functional, and computed tomographic characterization of idiopathic inflammatory myopathy-associated interstitial lung disease: a retrospective cohort study.”^1^ The study provides valuable insights into the epidemiological, clinical, and imaging features of this rare condition. Nevertheless, we would like to highlight several methodological and interpretive points that we believe could refine the conclusions. 

First, although lung biopsy is often unnecessary in cases with clear clinicoradiological concordance such as nonspecific interstitial pneumonia patterns, nearly half of the patients in the study presented with an indeterminate pattern. In such cases, histopathological confirmation is strongly recommended in order to improve diagnostic accuracy.^2^ The absence of biopsy in this large subgroup limits pathological characterization and may affect treatment decisions. 

Second, the cohort was predominantly composed of antisynthetase syndrome patients, with other idiopathic inflammatory myopathy (IIM) subtypes being underrepresented. This limits the generalizability of the findings to the wider IIM population. Moreover, no significant association was reported between antibody profiles and functional or respiratory outcomes. This could have provided additional prognostic information. 

Third, the time frame for assessing changes in FVC was not specified. In progressive fibrosing interstitial lung disease, a ≥ 10% annual decline is considered clinically meaningful and prognostically relevant. Without standardized intervals and clear thresholds, interpretation of FVC change becomes challenging-especially since FVC is effort-dependent and may decline due to muscular weakness rather than interstitial disease progression.^3^ The use of DL_CO_ could have provided a more reliable measure of interstitial involvement. 

Fourth, the diagnosis of pulmonary hypertension relied solely on echocardiography and radiological findings. The gold standard, right heart catheterization, was not performed, which may have resulted in underdiagnosis.^4^ The discussion statement “We found no cases of pulmonary arterial hypertension” is therefore methodologically uncertain and likely reflects the limitations of noninvasive assessment in patients with fibrotic lung disease. 

Fifth, it is unclear how HRCT scans were reviewed. The methods section suggests that they were evaluated by two of the authors of the study, but a consensus approach or interobserver agreement was not mentioned. Such details are important to ensure reproducibility. 

Finally, although the authors reported improvement in symptoms such as dyspnea and cough, this appears to have been based on patient self-report without validated scales. Given that treatment was individualized, the relationship between therapy and symptom or functional improvement remains difficult to assess. 

We commend the authors for assembling a well-characterized cohort and for providing long-term follow-up data. We hope that our considerations will encourage further research to strengthen diagnostic accuracy, standardize functional assessment, and refine prognostic evaluation in IIM-associated interstitial lung disease. 
