# Supplementary material for: CT characterization of idiopathic inflammatory myopathy-associated interstitial lung disease: frontiers to strengthen diagnostic accuracy
Source: J Bras Pneumol. 2025 Nov 19;51(5):e20250332. doi: 10.36416/1806-3756/e20250332 (PMC13143722; doi:10.36416/1806-3756/e20250332)
Supplement: Related texts

# Authors’ Reply

**Authors:** José Ricardo Bandeira de Oliveira, André Nathan Costa, Bruno Guedes Baldi, Mark Wanderley, Marcio Valente Yamada Sawamura, Ronaldo Adib Kairalla

**Affiliations:** 1. Divisão de Pneumologia, Instituto do Coração - InCor - Hospital das Clínicas, Faculdade de Medicina, Universidade de São Paulo - HCFMUSP - São Paulo (SP) Brasil.; 2. Instituto de Radiologia, Hospital das Clínicas, Faculdade de Medicina, Universidade de São Paulo - HCFMUSP - São Paulo (SP) Brasil.

We thank the authors for their thoughtful correspondence designated “CT characterization of idiopathic inflammatory myopathy-associated interstitial lung disease: frontiers to strengthen diagnostic accuracy,” which includes comments regarding our manuscript “Clinical, functional, and computed tomographic characterization of idiopathic inflammatory myopathy-associated interstitial lung disease: a retrospective cohort study,” as well as for the opportunity to clarify several methodological points. We herein explain the comments made by the authors point-by-point.

1. Lung biopsy in connective tissue disease (CTD)-related interstitial lung disease (ILD) is infrequently performed at our center because, in routine practice, histology usually does not modify management, especially when the diagnosis of a CTD is confirmed, even when there is an indeterminate pattern on CT scans. This approach-also acknowledged among the limitations of our study-reflects contemporary practice in autoimmune ILD.^1^ ^,^ ^2^
2. We agree with the authors that antisynthetase syndrome is over-represented in our cohort. This bias occurred due to convenience sampling in a referral ILD clinic, and inclusion criteria were restricted to participants with data available for longitudinal analysis in this retrospective design-points we have explicitly recognized as a selection bias limiting generalizability of the findings to the wider idiopathic inflammatory myopathy (IIM)-related ILD group. We did not evaluate the association of antibody profile with clinical and functional outcomes due to missing data regarding these serum exams.
3. We agree with the authors that the absence of DL_CO_ and respiratory muscle strength metrics (e.g., PI_max_/PE_max_) may influence interpretation of FVC. We decided not to apply the International Myositis Assessment & Clinical Studies (IMACS) or the current ATS/ERS recommendations that define clinically meaningful (e.g., ≥10%) annual decline, because annual, standardized spirometry results were not consistently available due to the retrospective nature of the study. We therefore reported change between the first and last available tests and recognize the effort-dependence of FVC as a limitation.
4. We agree that right-heart catheterization remains the gold standard for confirming pulmonary hypertension (PH). In our cohort, however, this invasive test was not performed due to the clinical judgment of the health care team, mainly because it was considered unlikely to change management decisions or was limited by the functional status of the patients. This reflects the inherent constraints of a retrospective study and underscores some of its methodological limitations. In line with the 2022 ESC/ERS pulmonary hypertension guideline,^3^ our surveillance used recommended screening tools-transthoracic echocardiography and CT measures (e.g., main pulmonary artery diameter/PA:A ratio). Although suspected PH was noted by echocardiography in a subset of patients, we found no features suggesting group-1 PH (pulmonary arterial hypertension; PAH); most suspicions were compatible with PH associated with parenchymal lung disease (group 3).
5. HRCT examinations were reviewed by two thoracic radiologists with expertise in ILD, and, in cases of disagreement, adjudicated by an ILD pulmonologist-thereby ensuring a consensus-based final interpretation.
6. Symptom data necessarily relied on patient reports because of the retrospective chart-review design. Validated scales to assess symptoms were not routinely applied in our center. Dyspnea was graded using the mMRC scale, which was the instrument routinely applied in our service.

We appreciate the colleagues’ engagement and believe these clarifications strengthen the interpretation and context of our findings.

## References

[1] Kannappan R, Kumar R, Cichelli K, Brent LH (2024). A Review of Myositis-Associated Interstitial Lung Disease. J Clin Med.

[2] Johnson SR, Bernstein EJ, Bolster MB, Chung JH, Danoff SK, George MD (2024). 2023 American College of Rheumatology (ACR)/American College of Chest Physicians (CHEST) Guideline for the Screening and Monitoring of Interstitial Lung Disease in People with Systemic Autoimmune Rheumatic Diseases. Arthritis Care Res (Hoboken).

[3] Humbert M, Kovacs G, Hoeper MM, Badagliacca R, Berger RMF, Brida M (2022). 2022 ESC/ERS Guidelines for the diagnosis and treatment of pulmonary hypertension. Eur Heart J.
